# Hydrogen sulfide exposure in an adult male

**DOI:** 10.4103/0256-4947.59379

**Published:** 2010

**Authors:** Bassam Doujaiji, Jaffar A. Al-Tawfiq

**Affiliations:** From the ^a^Internal Medicine Services Division, Dhahran Health Center, Saudi Aramco Medical Services Organization, Saudi Aramco, Dhahran, Saudi Arabia

## Abstract

Hydrogen sulfide (H_2_S) is responsible for many incidents of occupational toxic exposure, especially in the petroleum industry. The clinical effects of H_2_S depend on its concentration and the duration of exposure. H_2_S is immediately fatal when concentrations are over 500-1000 parts per million (ppm) but exposure to lower concentrations, such as 10-500 ppm, can cause various respiratory symptoms that range from rhinitis to acute respiratory failure. H_2_S may also affect multiple organs, causing temporary or permanent derangements in the nervous, cardiovascular, renal, hepatic, and hematological systems. We present a case of occupational exposure to H_2_S leading to multi-organ involvement, acute respiratory failure, organizing pneumonia, and shock resembling acute sepsis. The patient also developed mild obstructive and restrictive pulmonary disease and peripheral neuropathy.

Hydrogen sulfide (H_2_S) is responsible for many incidents of occupational toxic exposure, especially in the petroleum industry. The clinical effects of H_2_S depend on its concentration and the duration of exposure. H_2_S is immediately fatal when concentrations are over 500-1000 parts per million (ppm).

Hence, H_2_S has been referred to as the “knock down gas” because inhalation of high concentrations can cause immediate loss of consciousness and death.^2^,^3^ However, prolonged exposure to lower concentrations, such as 10-500 ppm, can cause various respiratory symptoms that range from rhinitis to acute respiratory failure.

There are many cases of H_2_S exposure in the agricultural industry^4^ and their prevalence has increased markedly with the development of porcine confinement facilities.^1^,^5^ H_2_S is the primary chemical hazard of natural gas production.^6^ We report a severe case of hydrogen sulfide (H_2_S) intoxication. The patient survived long enough to observe the sequelae of this entity, which can include neuropsychiatric morbidity.

## CASE

The patient was a 31-year-old male who worked in an oil refinery. He was brought to the emergency department with fever (a temperature of 39.3°C) and respiratory symptoms. He was hypotensive with a blood pressure of 68/40 mm Hg. He reported that he had been welding in a large container used for the storage of sulfur compounds in an open space before the onset of symptoms. No other chemical compounds were used and the container was clean at that time, but there were some unknown fluid residues on the floor. At the beginning of the welding process, white fumes with a “rotten egg” odor emanating from the container. The patient immediately felt dizzy and developed rhinorrhea, teary eyes, nausea, and shortness of breath, chest tightness and cough. These symptoms increased over the following hours followed by hemoptysis. He was seen by first aid providers and was removed from the scene, given oxygen, and was transported to the emergency room. There were no other workers in the same place during the event of the poisoning. He was using his personal protective equipment, including gowns and a mask.

**Figure 1:**
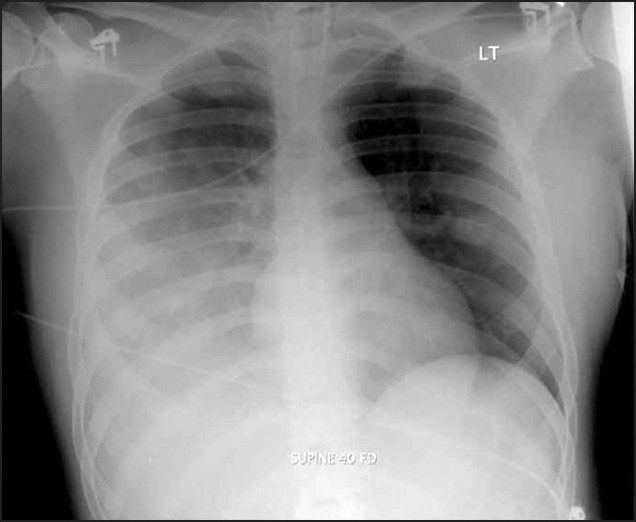
Chest x-ray on admission to ICU, showing right consolidation and pleural effusion.

Subsequently, the patient was admitted to the intensive care unit. On examination, he was found to be hypotensive (BP 68/40 mm Hg) and tachypneic (respiratory rate of 26/min). The neck was supple without any lymphadenopathy. A chest examination revealed bilateral rhonchi, but examination of the heart and abdomen found no abnormalities. Neurologically, he was combative and confused initially and became lethargic and obtunded later. There were no skin lesions. Shortly after admission, the patient developed acute respiratory failure requiring mechanical ventilation. Chest radiography (CXR) showed right pleural effusion and consolidation (Figure 1). Initial and subsequent laboratory (Table 1) revealed signs of ischemic cardiac injury, abnormal coagulation profile, renal insufficiency, and slight leukocytosis. Arterial blood gas showed a pH of 7.34; PCO_2_: 44 mm Hg; PO_2_: 77 mm Hg; and oxygen saturation: 95% on an inspired oxygen of 35%. Other laboratory data were as follows: BUN 43 mEq/L, creatinine 2.6 mg/dL, Na 135 mEq/L, K 4.8 mEq/L, Cl^−^ 105 mEq/L, and CO_2_ 19 mEq/L.

**Table 1:** The patient's laboratory data over the hospital course.

	Day 1	Day 2	Day 3 (ICU admission)	Day 4	Day 7
WBC (k/mm^3^)	14.7	1.5	1.6	8.5	22.9
Neutrophils %	88	21	8	16	70
Hemoglobin (g/dL)	13	12.7	13	12.4	11.5
Platelets (k/mm^3^)	289	237	223	202	294
BUN (mg/dL)	-	-	43	25	31
Creatinine (mg/dL)	-	-	2.6	1.1	0.9
INR (ratio)	-	-	1.9	1.3	1
PTT (seconds)	-	-	44	49	32
Troponin I (ng/mL)	-	-	37.6	9.4	<0.5
EKG	-	-	Inferior infarct	-	Normalized
Echocardiogram	-	-	EF 30%, impaired LV wall motion	-	EF>55%, LV function normalized

BUN=Blood urea nitrogen; INR=International ratio; PTT=Partial thrombin time; EKG=Electrocardiogram

As the patient was hypotensive, he was resuscitated with intravenous fluid and vasopressors. Intravenous hydrocortisone was started for chemical pneumonitis, but it was stopped after four days because there were no signs of improvement. Infections were ruled out and empirical broad-spectrum antibiotics were subsequently discontinued. All serological studies were negative, including *Mycoplasma*, *Legionella*, and HIV. A thoracentesis revealed an exudative pleural fluid. The gram stain showed many cells, 90% neutrophils, no organisms, and the cultures were negative. Cytology on bronchoalveolar lavage showed a few cells consistent with herpes simplex infection, thought to be a contamination from an upper airway and nasal infection. After several days of supportive care, the patient became hemodynamically stable with improved cardiac function and was extubated successfully.

However, he continued to have a right lower lobe consolidation (Figures 2a and 2b) despite appropriate antimicrobial therapy, including acyclovir. Lung biopsy via video-assisted thoracoscopic surgery was performed and showed diffuse alveolar damage with organizing pneumonia. Special stains for herpes viruses were negative. Sections of the lung showed the presence of numerous alveolar spaces lined by reactive pneumocytes type II. Many of the alveolar spaces were filled with an admixture of macrophages, scattered eosinophils, and neutrophils. In addition, several alveoli showed clumps of proliferating fibroblasts admixed with histiocytes and other inflammatory cells, denoting the presence of organizing pneumonia. He was restarted on intravenous hydrocortisone and showed a remarkable response with significant improvement in respiratory symptoms and radiographic findings (Figure 3).

**Figure 2a:**
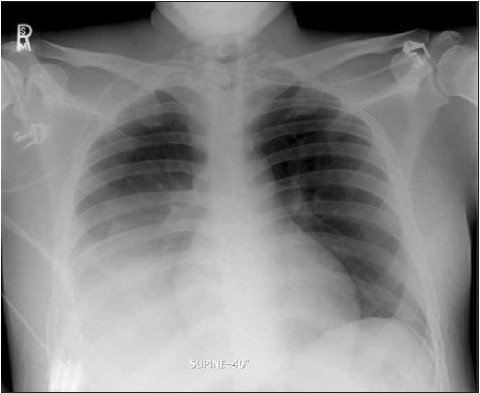
Chest x-ray three weeks after presentation, showing persistent right lower lobe consolidation.

**Figure 2b:**
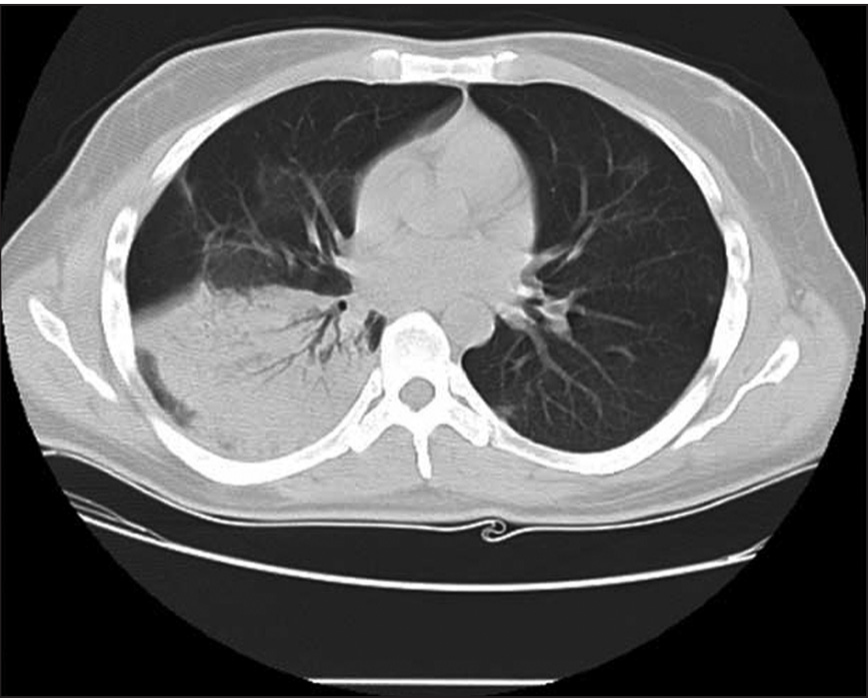
Chest CT scan, three weeks after presentation, showing persistent right lower lobe consolidation.

**Figure 3:**
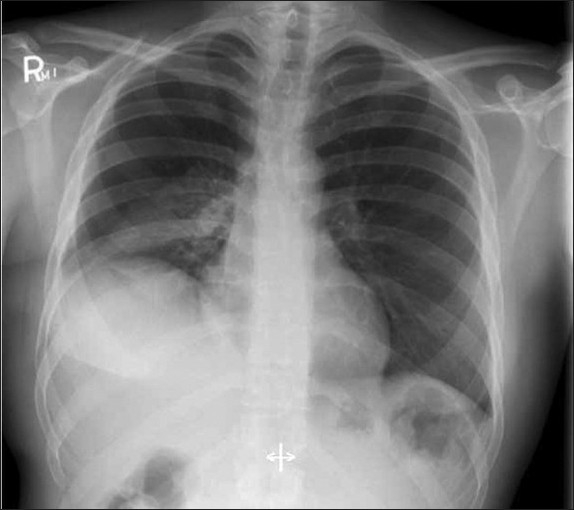
CXR, two days after initiating high dose steroids following lung biopsy, showing significant improvement in right lower lobe consolidation.

About 40 days after the incident, spirometery revealed a mild obstruction with an insignificant response to bronchodilators. Lung volume showed mild restriction and the diffusion capacity was at low-normal levels after correction for alveolar volume. The findings were consistent with mixed restrictive and obstructive pulmonary disease. He developed grayish nail-bed discoloration, suggestive of an exposure to a sulfur compound. Neurological evaluation, including an electromyography, revealed evidence of peripheral neuropathy. A follow-up chest x-ray after discharge showed complete resolution of his pulmonary infiltrate.

## DISCUSSION

Hydrogen sulfide (H_2_S) is the primary chemical hazard of natural gas production.^6^,^7^ In a retrospective review from oil and gas industry in Canada revealed 221 cases of H_2_S exposure from 1969 to 1973, and 173 patients were transported to the hospital; 14 victims (6%) were dead on arrival.^8^

The findings in this case are consistent with exposure to H_2_S. There was a history of an odor of rotten eggs emanating from residues in the work site that was suggestive of H_2_S with related symptoms and clinical findings, as described previously.^9^–^11^ H_2_S is a colorless gas with a characteristic odor.^12^ However, persistent exposure to air concentrations above 100 ppm produces olfactory fatigue, which impairs the ability to detect the characteristic odor of rotten eggs.^12^

Following the inhalation accident, the patient developed multi-organ involvement simulating sepsis: acute respiratory failure, obtundation, leucopenia, neutrophilia, abnormal coagulation profile, renal insufficiency, shock, cardiac injury, and reduced cardiac output (an ejection fraction of 30%).^9^,^13^ A persistent pulmonary infiltrate proved to be an organizing pneumonia. Organizing pneumonia is characterized by the presence of granulation tissue in the distal air spaces consisting of fibroblasts–myofibroblasts embedded in connective tissue.^14^ When organizing pneumonia is an associated feature, the term, “bronchiolitis obliterans” is added. Bronchiolitis obliterans organizing pneumonia (BOOP) may follow pulmonary infection, drug toxicity, or may appear in the context of connective tissue diseases or after lung or bone marrow transplantation.^15^ Cryptogenic organizing pneumonia (COP), the idiopathic form of organizing pneumonia (also known as idiopathic BOOP), is a distinct clinical entity. COP has the predominant features of pneumonia, rather than a primary airway disorder.^14^ The mainstay of treatment of BOOP is with a corticosteroid resulting in a rapid clinical improvement and clearing of the opacities on chest imaging without significant sequelae.^14^,^16^

Injury due to H_2_S exposure occurs primarily by inhalation. Once absorbed, the compound is distributed in the blood and taken up by the brain, liver, kidney, pancreas, and small intestines. Sulfur compounds are severely irritating to the respiratory tract, leading to rhinorrhea, sneezing, sore throat, wheezing, shortness of breath, chest tightness, hemoptysis, and a feeling of suffocation.^1^ Sulfur compounds can cause leucopenia and neutropenia,^9^,^13^ as well as cardiac injury with elevation of troponin I and creatine kinase.^9^,^10^ The mechanism of H_2_S toxicity is related to inhibition of oxidative phosphorylation, which causes a decrease in the available cellular energy. A phenomenon referred to as “knockdown” was reported in oil field workers to describe a sudden, brief loss of consciousness associated with amnesia, followed by immediate full recovery. This phenomenon usually occurs after short-term exposure to very high concentrations of H_2_S.^17^

Various pulmonary complications may follow inhalation injury. In a study of 203 patients with first- to third-degree burns, lung complications developed in 7.8%, leading to adult respiratory distress syndrome (ARDS) in 5.4%.^18^ Organizing pneumonia may occur following inhalation of toxic fumes and chemicals. These pathological changes are relatively well-known for patients who have been exposed to relatively high concentrations of any slightly water-soluble, toxic, inhaled compound (not unique to H_2_S) and may represent a spectrum of inhalation concentrations and severity. It is interesting to note that unilateral pulmonary abnormalities occurred in the present case. Similarly, in the study cited above, 36% of the patients had only right lung involvement.^18^ The presence of leucopenia and neutropenia in the present case is also interesting. In a study of chronic exposure to H_2_S, the absolute mean numbers of white blood cells, lymphocytes, and neutrophils were seen to be significantly decreased in the exposed group compared with the control.^19^

The patient showed evidence of neuropathy on follow-up visits. Annual neurological and neuropsychological testing for at least five years is recommended for patients with H_2_S exposure because of the potential chronic neurological sequelae.^20^ Other reports suggest that temporary memory loss, attention deficits, blunted affect, permanent retrograde amnesia, executive function deficits, slowing in central information processing, and planning deficits may occur in such patients.^17^

The mainstay of therapy is supportive care. There are reports that suggest early administration of hyperbaric oxygen, amyl nitrite, and sodium nitrite may be beneficial.^2^,^21^ Amyl nitrite-induced methemoglobinemia is due to competitive binding of the hydrosulfide anion. This effect presumably reactivates and protects cytochrome oxidase.^22^ However, one of the toxic effects of H_2_S is the inhibition of cytochrome oxidase. Nitrites produce methemoglobin which has a higher affinity for H_2_S than for cytochrome oxidase. The resulting sulfmethemoglobin eventually returns to hemoglobin. Hyperbaric oxygen in a few case reports and animal studies may work as an adjuvant treatment in patients with persistent neurological injury or oxygenation defects.^23^ Therapeutic red cell exchange may also be used to treat H_2_S toxicity similar to the treatment of aniline, arsine, chloramines, carbon monoxide, cyanide, and, methemoglobinemia.^24^

As H_2_S is a potential problem in the transport and storage of crude oil, preventive measures are extremely important in preventing lethal exposure to hydrogen sulfide toxicity. Personal protective equipment should include safety glasses, respiratory protection or equipment, and long-sleeved shirts.^25^ In addition, limiting exposure at the work place and the use of a personal safety gas detector may aid in the protection of employees working with potential gas exposure. In a retrospective analysis, 77 of the 80 deaths were thought to be potentially preventable with the use of an H_2_S alarm or portable meters.^26^

In conclusion, we report a case of an occupational exposure to H_2_S leading to acute respiratory failure, multi-organ involvement simulating sepsis, and organizing pneumonia. The diagnosis of hydrogen sulfide poisoning relies mainly on the clinical presentation and exposure. H_2_S poisoning may lead to persistent neuropsychiatric morbidity. The treatment remains generally supportive and amyl nitrites may be beneficial.
